# Variation in Microbiota and Chemical Components Within *Pinus massoniana* During Initial Wood Decay

**DOI:** 10.3390/microorganisms13081743

**Published:** 2025-07-25

**Authors:** Bo Chen, Hua Lu, Feng-Gang Luan, Zi-Liang Zhang, Jiang-Tao Zhang, Xing-Ping Liu

**Affiliations:** Provincial Key Laboratory of Conservation Biology, School of Forestry, Jiangxi Agricultural University, Nanchang 330045, China

**Keywords:** *Pinus massoniana* Lamb., driving factors, metabolites, mycobiota, initial wood decays

## Abstract

Deadwood is essential for the forest ecosystem productivity and stability. A growing body of evidence indicates that deadwood-inhabiting microbes are effective decomposition agents, yet little is known about how changes in microbial communities during the initial deadwood decay. In a small forest area, we performed dense sampling from the top, middle, and bottom portions of two representative *Pinus massoniana* cultivars logs to track deadwood xylem microbiota shift during the initial deadwood decay. We found xylem mycobiota varied dramatically during the initial deadwood decay. Deadwood microbes might largely originate from the endophytic microbes of living trees during the initial deadwood decay. Notably, bark type is an important driving factor for xylem mycobiota changes during the initial deadwood decay. Ten upregulated metabolites were screened out by a univariate analysis approach. Moreover, our correlation analysis suggests that enriched microbes at class level was significantly correlated with the upregulated metabolites during the initial deadwood decay. Our work provides new insights into the process of mycobiota and metabolite changes during the initial deadwood decay.

## 1. Introduction

Deadwood is an important component in forest ecosystems, closely related to biodiversity, soil fertility, and spatial structure within forests [1,2,3]. Deadwood can be degraded by few organisms due to its formidable chemical and biological barrier, with fungi being the most significant participants in deadwood decomposition [4]. A growing body of experimental evidence indicates that various biotic and abiotic factors manipulate deadwood decomposition [5]. One of the most essential factors influencing the wood decay rate seems to be which fungi are first to colonize fresh deadwood [6]. Deadwood microbiota during the initial decay is frequently disturbed by its physicochemical properties, which are largely inherited from living trees [4,7]. In addition, fungi, compared with bacteria, remain the primary decomposers and main content for an extended period following tree mortality (0–15 years) [8]. Therefore, several previous studies focus on the mechanism of mycobiota establishment during the initial wood decay [4,9,10].

The establishment of deadwood microbiota is a dynamic and directional process [11]. The initial deadwood microbiota is similar to endophytes within living trees, becoming more decay-specific with increasing degrees of decay. Current evidence supports that the microbes within living trees can become an important origin of deadwood microbiota under certain conditions. A study has confirmed, through culturing regimes, that some wood-decay fungi can exist latently in living trees [12]. Subsequently, other researchers have supported this conclusion by measuring fungal DNA fragments in living trees [13]. However, little is known about how fungal communities vary during the initial decay. Bark retention status on deadwood is one of the important indices in assessing decay stages [11]. The impact of bark on the deadwood microbial community is mainly through two ways: it acts as a barrier for trees to resist microbial invasion or a regulator of the deadwood microenvironment [14,15,16]. Notably, the actions of bark on deadwood microbiota are unpredictable to a large extent [14]. For instance, bark looseness might stimulate the establishment of wood-decay fungi due to an increase in fauna [4,17]; instead, it may inhibit fungi establishment due to a decrease in moisture [18]. However, these studies were carried out in different tree species or a wide range of areas, making it difficult to control conditions. Little is known about how various types of bark influence deadwood microbiota within the same tree species. In addition, previous studies have confirmed that some microbes represented in deadwood can live in local soil, suggesting that deadwood fungi may originate from the soil [19]. However, the influence of soil on wood microbiota is notably complicated, with some research results being contradictory [9,10,20]. This disparity is undoubtedly partially attributed to the technical constraints and partly to wood characteristics. Therefore, it is essential to supplement the foundational data for the mechanism of wood–soil interaction by exploring unstudied areas, including unexplored climatic conditions and a broader range of tree species.

A relatively wide of studies focus on the microbe–metabolite interaction within living trees rather than deadwood–metabolite interaction. Previous studies have identified many metabolites from the organs of pine trees [21,22], such as plant hormones, amino acid-related compounds, phenolic compounds, etc. Dynamic metabolites are regarded as a bridge between plants and microbiota [23]. Wood-decay fungi can strengthen their niche occupancy abilities by releasing soluble metabolites [24]. At present, we still know very little about the association between the deadwood microbiota and metabolites during the initial decay.

In southern China, Masson pine (*Pinus massoniana* Lamb) is one of the most important native conifer trees, which adapts to acidic infertile soils [11,25]. Masson pine can be divided into two groups (thin-barked and thick-barked types) according to morphological structures. The tortoiseshell-shaped bark on thick-barked Masson pines is brown, with many cracks and fissures. In contrast, the scaly bark on thin-barked Masson pines is brownish-yellow [26]. In this study, taking advantage of the essential gap in the literature, we focus on Masson pines (*Pinus massoniana* Lamb), a locally important tree. A field trial was performed to explore the microbial communities and metabolite shifts during the initial *Pinus massoniana* decay. We aimed to analyze the effects of bark types and vertical positions on xylem microbiota within *Pinus massoniana* deadwood. In addition, we explored the association between microbes and metabolites in *Pinus massoniana* deadwood. Our findings provide valuable insights into changes and driving factors of mycobiota during the initial *Pinus massoniana* decay.

## 2. Material and Methods

### 2.1. Study Site

The field trial was conducted in Meiling National Forest Park, Nanchang, Jiangxi province, China, from May 2023 to September 2023. The local average annual temperature was 17 °C, the average annual humidity was 75%, the average annual total precipitation was 676 mm, and the soil was subtropical red soil. The field site (altitude: 248.52 m, latitude: 28.76° N, longitude: 115.80° E) used in this study was located within a mixed forest stand, planted with *P. massonian* Lamb, Chinese guger tree *Schima superba* Gardner & Champ., and Slash pine *P. elliottii* Engelm. Masson pine was the dominant species in this mixed forest stand. The average height of all pine trees exceeded 10 m and the understory plant species included a small amount of shrubs, such as *Loropetalum chinense* (R. Br.) Oliv. and *Eurya muricata* Dunn. The south-facing slope was selected in this study area at approximately 20 degrees.

### 2.2. Deadwood Preparation

*Pinus massoniana* trees were classified into two types (thin-barked and thick-barked pines) following the criteria of the Forestry Department of Anhui Agricultural College (1982) [26]. We felled ten healthy *Pinus massoniana* trees (15 cm DBH), including five thin-barked and five thick-barked pines, at intervals of at least 10 m along a 150 m transect. The bottom section of each pine was cut into one log, each 1.7 m in length. Then, the cut surfaces on both ends of each pine log were coated with tree wound cream (Shandong Haidai Greenland Bioengineering Co., Ltd., Jinan, China) to avoid rapid moisture loss. To simulate the decomposition of standing deadwood, the bottom of all logs was inserted into a small pit that was 20 cm deep and filled with soil to ensure these logs stood firmly.

### 2.3. Sample Collection from Deadwood

The xylem samples were collected from each log at two decay time points: on the initial day when the pines were felled, and on the 120th day when the bark began to fall naturally from the logs. The top (30 cm from the upper end), middle, and bottom (30 cm above the ground) of each log were selected as sampling positions. Each sampling position was divided into four sampling points which were evenly distributed around the circumference to eliminate the differences in microbes in different directions of deadwood. Before the beginning of sampling, an alcohol-washed chisel was used to remove an approximately 1 cm^2^ section of bark for each sampling point to expose the xylem. Then, an increment borer (d = 5.15 mm, Hangzhou Lvbo Instrument Co., Ltd., China, Hangzhou, China) was used to extract cores with lengths of 27 mm from each sampling point of pine logs. Finally, the sampled drill holes were rapidly sealed with an autoclave-sterilized wooden dowel. To control the microbial contamination, the chisel and increment boring device were rinsed with 75% alcohol and distilled water, respectively, and allowed to dry naturally after each sampling. The subsamples from four sampling points in the same sampling position were mixed into one sample, which was subsequently transferred into a 20 mL sterilized test tube. All samples were transported to the laboratory using dry ice and then stored in a −80 °C freezer until DNA and metabolites extraction.

### 2.4. DNA Extraction, PCR Amplification, and Sequencing

Total xylem genomic DNA was extracted from sixty samples using the conventional cetyltrimethylammonium bromide (CTAB) method. DNA integrity and size were monitored by 1% agarose gel electrophoresis. Then, DNA was diluted to 1 ng/µL through sterile water according to DNA concentration. Internal Transcribed Spacer (ITS)-based amplification was carried out using specific primers (ITS3-2024F: 5’-GCATCGATGAAGAACGCAGC-3’; ITS4-2409R: 5’-TCCTCCGCTTATTGATATGC-3’) directionally targeting hypervariable regions (ITS3-2024F and ITS4-2409R) of ITS genes [27]. Furthermore, gene products were appended with forward and reverse error-correction barcodes to identify each sample sequence accurately and derive precise taxonomic information. After the PCR reaction, equal volumes of 1× Tris-Acetate-EDTA (TAE) buffer with the PCR products were mixed and conducted electrophoresis on a 2% agarose gel to detect the PCR products. Finally, amplicons (PCR products) were quantified, following which the normalized equimolar concentrations of amplicons were pooled and sequenced on an Illumina NovaSeq 6000 platform. NovaSeq Control Software v1.7 was used for real-time data processing. The FASTQ files were obtained from above steps and used to analyze the diversity, composition, and variation of the microbial communities during the early stage of pine deadwood decomposition.

### 2.5. Sequence Access

The nucleotide sequences of the ITS region of different fungal isolates are available from the National Center for Biotechnology Information Sequence Read Archive under BioProject PRJNA1185929.

### 2.6. Metabolome Determination

The metabolites that occurred in the xylem samples were extracted and meticulously analyzed by Wekemo Technology Group Co., Ltd., Shenzhen, China, utilizing metabolomic techniques based on Liquid Chromatography-Tandem Mass Spectrometry (LC-MS/MS). Briefly, 100 mg of deadwood tissues were pulverized with liquid nitrogen, and 500 μL of chilled 80% methanol was added. The mixture was fully vortexed, incubated on ice for 5 min, and then centrifuged at 15,000× *g* for 20 min at 4 °C to obtain the resulting supernatants. Ultra-high performance liquid chromatography coupled to tandem mass spectrometry (UHPLC-MS/MS) analyses were carried out using a Vanquish UHPLC system (Thermo Fisher Scientific, Waltham, MA, USA) and coupled with an Orbitrap Q ExactiveTM HF-X mass spectrometer (Thermo Fisher Scientific, USA). The resulting supernatants were injected into the LC-MS/MS system with a Hypersil Gold column (100 × 2.1 mm, 1.9 μm) using a 12 min linear gradient at a flow rate of 0.2 mL/min. The solvent gradient was set as follows: 2% B, 1.5 min; 2–85% B, 3 min; 85–100% B, 10 min; 100–2% B, 10.1 min; 2% B, 12 min [28]. The qualitative and quantitative results were exported as a table that allows us to analyze the composition, variation, and KEGG pathways of metabolites within the early stage of decomposition of Masson pine deadwood.

### 2.7. Statistical Analysis

To ensure the accuracy of the ASV (Amplicon Sequence Variant) cluster and subsequent analysis, the raw sequencing data were meticulously processed and filtered to obtain valid and reliable data. Specifically, raw data FASTQ files were imported into QIIME 2 (Version 2021.2), following which demultiplexed sequences from each sample were effectively filtered, trimmed, de-noised, and merged [29,30]. Then, the chimeric sequences were identified and eliminated using VSEARCH (Version 2.7.0), a QIIME 2 plugin, ensuring the accuracy of the ASV table by clustering effective tags with a similarity threshold of 0.97. Furthermore, USEARCH (Version 11.0.667) was utilized to align ASV sequences to a pre-trained RDP (Ribosomal Database Project) or database to generate draft taxonomy tables. Subsequently, to refine these tables, any mention of contaminating mitochondrial and chloroplast sequences was removed through a series of text-processing commands executed within the Linux environment, resulting in final taxonomy tables. The raw data files of metabolome obtained were introduced into Proteowizard (Version 3.0.8789) to transform the file format for subsequent analysis, then subjected to peaks identification, filtration, and alignment for each of these metabolites utilizing XCMS R packages (Version 3.1.3). Furthermore, peak intensities were normalized to the total spectral intensity and mapped to mzVault, MassList, and mzCloud (https://www.mzcloud.org, accessed on 1 February 2024) databases.

All statistical calculations were conducted with R packages (Version 3.6.2, https://www.rproject.org). To compare the difference in the fungal communities between different decay time points, sampling positions, and bark types, one-way analysis of variance (ANOVA) followed by the least significant difference (LSD) test was used to analyze the alpha diversity indices (including Shannon–Wiener index, Simpson index, and Pielou index) of the microbial communities. These differences were further analyzed using Kruskal–Wallis pairwise comparisons to determine the exact *p*-value. The ASVs’ abundance in the microbial communities was transformed into a Bray–Curtis distance matrix, and the similarity and dissimilarity were visualized using principal coordinates analysis (PCoA). Subsequently, these differences among microbial communities were computed through analysis of dissimilarity (Adonis). The ASV clusters among all groups were constructed and presented as Venn diagrams to compare their compositional variations. The indicator species in deadwood microbes were compared across different groups using Wilcoxon rank sum tests based on ASVs with median relative abundance from each sample > 0.001%, and corresponding *p*-values were adjusted for multiple tests using false discovery rate (FDR) set at 0.05. Furthermore, Pearson’s coefficients were used to examine the co-occurrence relationships among microbes at class and genus levels. *p* < 0.05 was considered statistically significant.

The differences in metabolic profiles were analyzed through principal component analysis (PCA), and these differences among microbial communities were computed through analysis of dissimilarity (Adonis). Upregulated or downregulated metabolites were selected through univariate analysis based on fold change and *t*-test (|log2(FC)| > 2, *p*-value < 0.05). To clarify the hierarchical level of biological function related to metabolites in deadwood, Kyoto Encyclopedia of Genes and Genomes (KEGG) pathway enrichment analysis was performed using tools available in the KEGG database (release 95.2, https://www.kegg.jp). Then, the relationships were determined between microbes and upregulated or downregulated metabolites using the Pearson correlation coefficient test to understand the interactions between metabolites and microbes in the early decomposition.

## 3. Results

### 3.1. Fungal Community Changes Between Living Pinus massoniana and Pinus massoniana Deadwood

**Figure 1:**
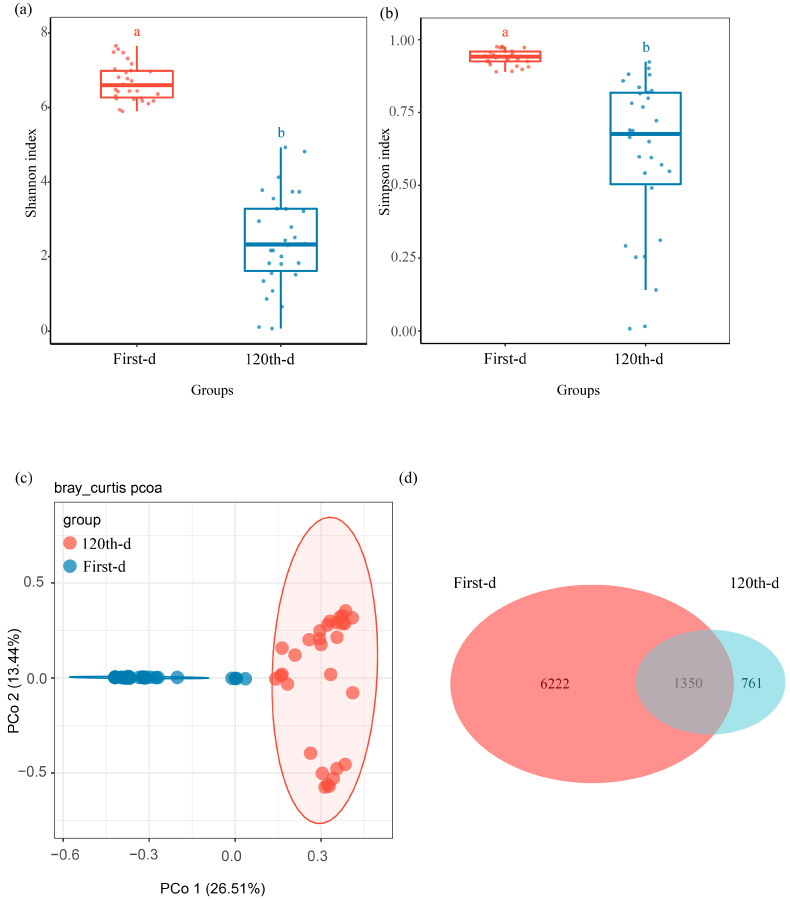
Community composition and variation in the xylem fungal community at decay time points. Comparison of (**a**) Shannon diversity index, and (**b**) Simpson diversity index of xylem fungi between the two decay time points. (**c**) Difference in xylem fungal community structure between the two decay time points at the ASV level. (**d**) Venn diagram showing shared and unique ASVs of xylem fungi between the two decay time points, with an overlapping region indicating common ASVs.

**Figure 2:**
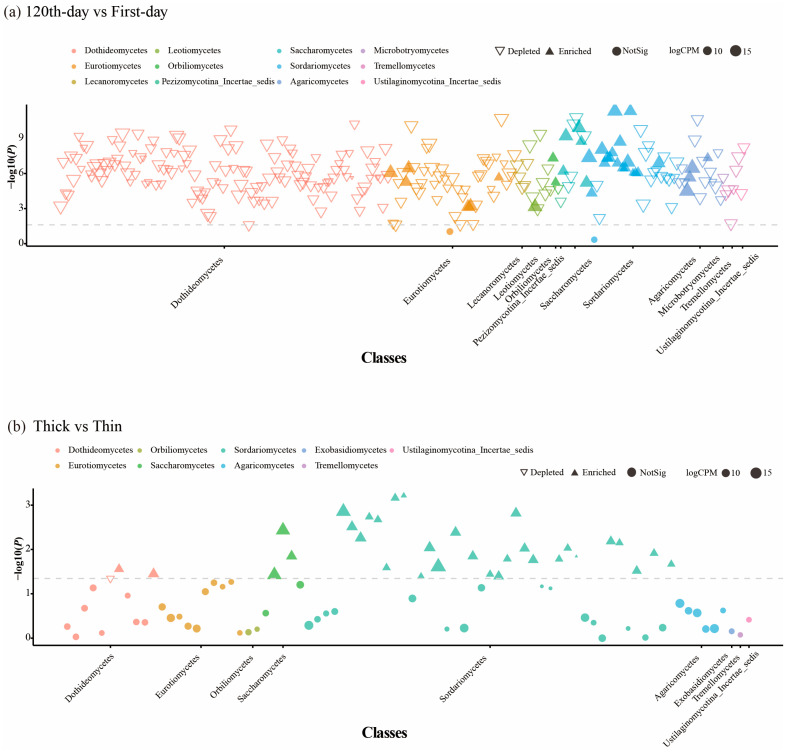
Manhattan plots of fungal ASVs enriched or depleted in the thick-barked group vs. the thin-barked group or the 120th-day group vs. the first-day group. Each dot or triangle represents a single OTU. ASVs enriched in the thick-barked group or thin-barked group are represented by filled triangles or empty triangles, respectively. The dashed line corresponds to the false discovery rate-corrected *p*-value significance threshold (*p* < 0.05). The color of dots corresponds to the class level of each ASV, and the size of dots corresponds to the relative abundance of that ASV in the xylem sample. CPM, counts per million.

To explore the fungal community changes during the initial *Pinus massoniana* decay, the mycobiota of the first-day and 120th-day groups were compared. The Shannon diversity index (*H* = 44.26, *p* = 2.872 × 10^−11^) and Simpson diversity index (*H* = 42.32, *p* = 7.762 × 10^−11^) were significantly different between the first-day and 120th-day groups (Figure 1a,b and Figure S1a,b). Our PCoA based on Bray–Curtis distances showed a significant separation between samples for the two groups (*p* = 0.001, *R^2^* = 0.24, Figure 1c). *Agaricomycetes* and *Sordariomycetes* were the dominant class in the 120th-day group; however, *Dothideomycetes* was the dominant class in the first-day group (Figure S2a). A notable overlap was detected in both groups: 1350 ASVs accounted for 17.83% in the first-day group and 63.95% in the 120th-day group (Figure 1d). Moreover, the Manhattan plot showed that ASVs of xylem fungi enriched in the 120th-day group belonged to *Agaricomycetes*, *Eurotiomycetes*, *Orbiliomycetes*, *Leotiomycetes*, *Sordariomycetes*, and *Saccharomycetes* (FDR-adjusted *p* < 0.05) (Figure 2a).

### 3.2. Fungal Community Differed Between Thin-Barked and Thick-Barked Pinus massoniana Deadwood

**Figure 3:**
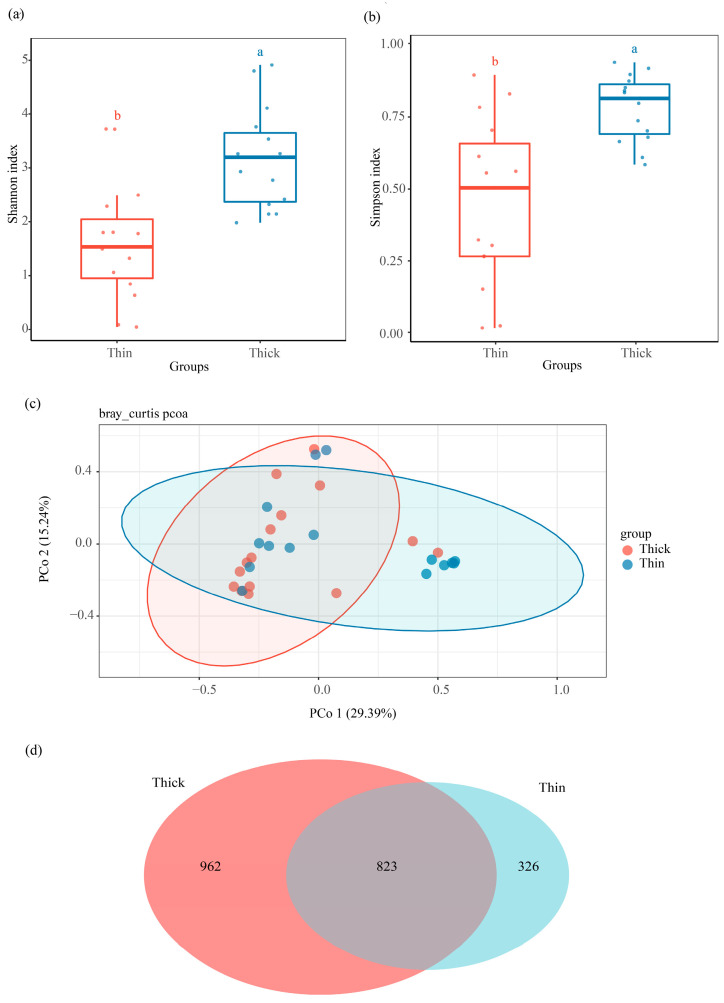
Community composition and variation in xylem fungal community with two deadwood types. Comparison of (**a**) Shannon diversity index, and (**b**) Simpson diversity index of xylem fungal community between the two deadwood types. (**c**) Difference in xylem fungal community structure between the two deadwood types at the ASV level. (**d**) Venn diagram showing shared and unique ASVs of xylem fungi between the two deadwood types, with an overlapping region indicating common ASVs.

To clarify fungal community variations in between thick- and thin-barked *Pinus massoniana* deadwood, xylem samples from two types of deadwood were collected and analyzed. The Shannon diversity index (*H* = 11.71, *p* = 0.0006) and Simpson diversity index (*H* = 10.87, *p* = 0.00098) were significantly different between the thick-barked group and thin-barked group (Figure 3a,b and Figure S1c,d). For PCoA results based on Bray–Curtis distances, significant differences were found in the fungal community between the thick-barked group and thin-barked group (*p* = 0.031, *R*^2^ = 0.072, Figure 3c). *Agaricomycetes* and *Sordariomycetes* were the dominant class in both thick-barked and thin-barked groups (Figure S2b). There were 823 ASVs which overlapped between these two groups, accounting for 46.11% in the thick-barked group and 71.63% in the thin-barked group, respectively. The number of unique ASVs in the thick-barked group was considerably more than that in the thin-barked group (Figure 3d). The Manhattan plot showed that ASVs enriched in the thick-barked group belonged to fungal classes, including *Dothideomycetes*, *Sordariomycetes*, and *Saccharomycetes* (FDR-adjusted *p* < 0.05) (Figure 2b). Furthermore, some ASVs enriched in the thick-barked group belonged to several genera, including *Cyberlindnera*, *Kuraishia*, *Nectria*, and *Pestalotiopsis* (FDR-adjusted *p* < 0.05) (Figure 2b, Table S1).

### 3.3. Fungal Community Differed Across Vertical Positions in Pinus massoniana Deadwood

**Figure 4:**
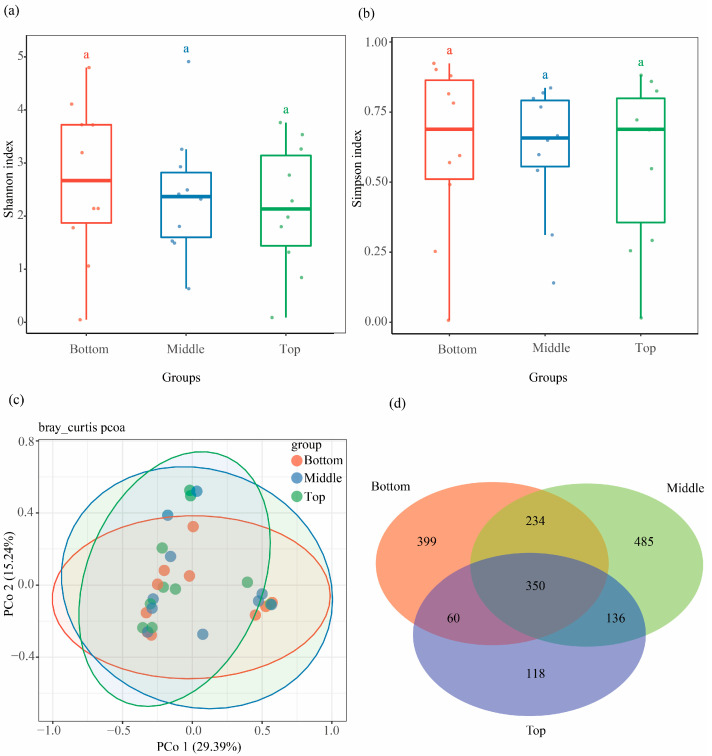
Community composition and variation in xylem fungal community with sampling positions. Comparison of (**a**) Shannon diversity index, and (**b**) Simpson diversity index of xylem fungi among three sampling positions. (**c**) Difference in xylem fungal community structure among three sampling positions at the ASV level. (**d**) Venn diagram showing shared and unique ASVs of xylem fungi among three sampling positions, with an overlapping region indicating common ASVs.

To examine variation in fungal community along the vertical positions of standing deadwood, xylem samples from different heights (bottom, middle, and top portions) were collected and analyzed. No significant difference was found in Shannon (*H* = 0.56, *p* = 0.7558) and Simpson (*H* = 0.16, *p* = 0.9219) indexes among the three sampling portions (Figure 4a,b and Figure S1e,f). PCoA results showed that the fungal community had no significant difference among the three sampling portions (*p* = 0.916, *R*^2^ = 0.044, Figure 4c). *Agaricomycetes* and *Sordariomycetes* were the dominant classes among the three sampling portions (Figure S2c). Only 399, 485, and 118 unique ASVs were detected in samples from the bottom, middle, and top portions, respectively (Figure 4d).

### 3.4. Wood Decomposition Metabolites and Their Correlation with Fungal Communities

**Figure 5:**
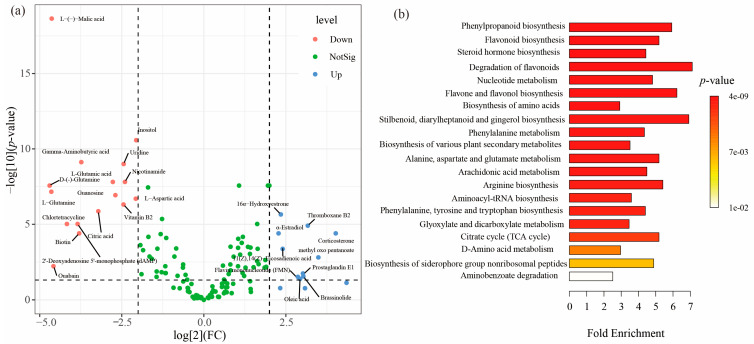
The variable characteristics of xylem metabolites during the early stage of decomposition. (**a**) Volcano plot showing the differentially accumulated [log_2_ (FC) on x-axis] and significantly changed [−log_10_ (*p*-value) on y-axis] metabolites in the first-day and 120th-day groups. Up means a metabolite being significantly upregulated in the 120th-day group (log_2_ (FC) > 2, −log_10_ (*p*-value) > 1.301). Down means a metabolite being significantly downregulated in the 120th-day group (log_2_ (FC) < −2, −log_10_ (*p*-value) > 1.301). (**b**) Over-representation analysis (ORA) enrichment analysis. The metabolic pathways with significant enrichment of the early stage of decomposition. The x-axis shows the enrichment ratio. The *p*-value is color-coded as the brightness, as shown in the legend.

Principal component analysis (PCA) showed that the metabolic profiles partially overlapped between the thick-barked group and thin-barked group (*p* = 0.162, *R*^2^ = 0.050, Figure S3b), and largely overlapped among the bottom, middle, and top groups (*p* = 0.651, *R*^2^ = 0.058, Figure S3c). However, the metabolic profiles completely separated between the first-day group and 120th-day group (*p* = 0.001, *R*^2^ = 0.331, Figure S3a). The differential accumulated and significantly changed metabolites in the first-day and 120th-day groups are shown in Figure 5a. In the first-day group, a higher abundance of sixteen metabolites were screened out, including antibiotics (Chlortetracycline), carbohydrates (Inositol), nucleic acids (Uridine, Guanosine, dAMP), organic acids (L-(-)-Malic acid, Citric acid), peptides (L-Glutamine, Gamma-Aminobutyric acid, D-glutamine, L-Glutamic acid, L-Aspartic acid), steroids (Ouabain), and vitamins and cofactors (Nicotinamide, Biotin, Vitamin B2). In contrast, ten abundant metabolites were selected in the 120th-day group, including hormones and transmitters (16α-Hydroxyestrone), lipids (Oleic acid, Prostaglandin E1, Thromboxane B2, 11(Z),14(Z)-Eicosadienoic acid), organic acids (methyl oxo pentanoate), steroids (Corticosterone, Brassinolide, α-Estradiol), and vitamins and cofactors (Flavin mononucleotide). Pathway enrichment analysis revealed that new tree death elicited alteration in metabolic pathways related to phenylpropanoid biosynthesis, flavonoid biosynthesis, steroid hormone biosynthesis, degradation of flavonoids, and so on (Figure 5b).

The correlation analysis between microbes and wood decomposition metabolites was shown in Figure S4. At the fungal class level, *Agaricomycetes* and *Sordariomycetes* were positively correlated with Thromboxane B2 and Corticosterone in upregulated metabolites, but negatively correlated with all downregulated metabolites. *Agaricomycetes* was also positively correlated with Flavin mononucleotide (FMN), 16α-Hydroxyestrone, and α-Estradiol in upregulated metabolites. *Sordariomycetes* was also positively correlated with Oleic acid, Brassinolide, 11(Z),14(Z)-Eicosadienoic acid, and methyl oxo pentanoate in upregulated metabolites. In contrast, *Dothideomycetes* was significantly positively correlated with all downregulated metabolites except Ouabain, but negatively correlated with 16α-Hydroxyestrone, α-Estradiol, 11(Z),14(Z)-Eicosadienoic acid, methyl oxo pentanoate, Thromboxane B2, Corticosterone in upregulated metabolites.

## 4. Discussion

### 4.1. Mycobiota Dynamics During the Initial Pinus massoniana Decay

In this study, mycobiota in living trees and deadwood were analyzed to explore the initial shift process of decay microbes. As shown in Figure 1a,b, the fungal diversity from fresh pine logs was quite high, which might be due to the effect of tree species and local environments [20]. For example, under uniform sampling conditions, and with similar tree ages and diameters at breast height (DBH), certain specimens of *Pinus koraiensis* exhibit Shannon–Wiener indices nearing a value of seven, whereas the minimal Shannon–Wiener indices observed in *Picea jezoensis* samples approximate a value of one [31]. Our results also show that lower fungal diversity index (Figure 1a,b) was one of the response acts to wood environmental changes.

The phylum *Basidiomycota* possesses great abilities to degrade lignin, and the phylum *Ascomycota* exhibits higher adaptability in metabolizing a wide range of plant residues and litter [32]. Thus, *Basidiomycota* and *Ascomycota* are most abundant in nearly all decay levels of pine wood [11]. We found that *Agaricomycetes* (*Basidiomycota*) and *Sordariomycetes* (*Ascomycota*) were more significantly abundant in the 120th-day group than in the first-day group (Figure S2a). However, only four ASVs belonging to *Agaricomycetes* were significantly enriched from first day to 120th day whereas 13 ASVs were significantly depleted over the same period (Figure 2a). The results indicate that the relative abundance increase in *Agaricomycetes* in the 120th-day group is attributed to the abundance growth of a minority of species within this phylum, which may have better adapted to the environment of decaying wood in the initial decay stage. We hypothesize that some species of wood pathogenic fungi (*Agaricomycetes*) lost their original hosts, while others, being wood-rotting fungi, failed to compete successfully due to highly overlapped ecological niches, leading to their depletion [33,34,35]. However, the underlying mechanisms are not fully elucidated. Further work with deadwood fungal species identification and function might help us understand deadwood microbiota shifts.

In nature, one mystery is the origin of the microbiota in deadwood. More than sixty percent of fungal ASVs were shared between the first-day group (fresh pine logs) and 120th-day group (Figure 1d), indicating that many wood-decay fungi can exist in living trees. This result supports the hypothesis that fungi in deadwood originate from living trees to a large extent [36,37]. Nonetheless, it is extremely challenging to conclusively assess the shared microbiota mentioned above, some of which might be contributed by air, soil, etc., and to differentiate tree-derived from shared microbiota without an isotope tracing study, which is difficult to determine through statistics. Further study will focus on determining the microbiota shift pathway with isotope-labeled methods.

### 4.2. Effect of Bark Types on Mycobiota Within Pinus massoniana Deadwood

The variations of bark types in living trees suggest different strategies against threats (insects, fungi, bacteria, fire, etc.) from surrounding environments [38]. However, little attention has been paid to the role of bark in wood after tree death. Masson pine, a native tree species in the south of China, can be traditionally categorized into two distinct types, thick-barked and thin-barked pine, based on the phenotypic characteristics of their bark. In this study, we test the effect of two deadwood types on mycobiota by sampling xylem from two types of Masson pine. Our results showed that the structural characteristics of mycobiota in the xylem varied significantly across different bark types (Figure 3a–c), indicating that Masson pine bark participates in the xylem fungal community establishment.

Essentially, the influence of bark on the mycobiota in deadwood is mainly manifested in two key aspects: a biological barrier to fungal invasion and a regulator of microenvironments. The various physical traits of bark serve distinct roles in defense against exogenous fungi; for example, a thick bark reduces the accessibility of the xylem to exogenous fungi compared with a thin bark [15]. Bark creates favorable microenvironments for mycobiota through nutrition, moisture, etc. [14]. The number of unique ASVs in the thick-barked group is much higher than in the thin-barked group (Figure 3d). Referring to Figure 3a–d, in the case of stronger biological barriers, the thick-barked pine still had more abundant fungal ASVs and higher diversity, indicating that the microenvironment created by the bark played a more important role in shaping the microbiota during the initial *Pinus massoniana* decay.

As shown in Figure 2b and Table S1, ASVs belonging to genera *Nectria*, *Kuraishia*, *Pestalotiopsis,* and *Cyberlindnera*, representing three classes (*Dothideomycetes*, *Sordariomycetes*, and *Saccharomycetes*), were significantly enriched in the thick-barked pines compared to the thin-barked pines. Previous studies have shown that the three genera are highly associated with wood decay [36,39,40]. *Nectria* is a wood-inhabiting genus commonly found in bark tissue, progressing from weak pathogenic invaders to saprotrophic decomposers of wood [41,42,43]. Specific *Nectria* species secrete xylanases to hydrolyze xylan, a major component of hemicellulose in plant cell walls [44]. Concurrently, multiple studies have validated that *Cyberlindnera* strains exhibit significant xylanolytic activity and cellobiose assimilation capabilities [39]. In contrast, research on the unique biological characteristics of *Kuraishia* remains scarce, with only fragmentary literature available. Existing studies suggest that certain species within this genus possess cellulose-utilizing potential, while others demonstrate robust nitrate-assimilating capacity [45,46]. However, it is extremely challenging to speculate how the enrichment of certain genera influences deadwood decomposition due to the uncertainty of fungal relationships. For example, the competitive relationships among fungi may inhibit the decay rate of organic matter [47]. Therefore, further research is needed to explore direct or underlying relationships among fungi.

### 4.3. Effects of Vertical Position on Mycobiota Community Variation in Pinus massoniana Deadwood

The microbial communities of deadwood near-ground sections are susceptible to soil due to direct contact with the ground, compared with far-ground points of deadwood [37,48,49]. Some studies demonstrated that xylem microbial communities exhibit minimal similarity to microbes in other plant tissues [20]. The thinner bark in the upper trunk and the thicker bark at the bottom might induce the variation in pine wood microbiota. Therefore, studying microbiota in the different parts of deadwood is crucial to fully understanding deadwood microbiota establishment. Our results showed that the average alpha diversity indexes were very similar among xylem samples collected at different heights above ground from pine deadwood, with no significant differences in the structural characteristics of their fungal communities (Figure 4a–c). However, a previous study has shown that mycobiota structure in different log sections (bottom, middle, top) exhibits significant differences, which is inconsistent with our findings [4]. This phenomenon may be attributed to two key factors, such as the relatively young age of the tree and variation in tree species characteristics. In this study area, we observed homogeneous bark thickness distribution along the main trunk of *Pinus massoniana*, a phenotypic trait potentially associated with rapid growth strategies in early developmental stages [50]. Furthermore, narrow cell lumina and limited numbers of bordered pits passively hamper fungi colonization in coniferous trees’ deadwood [51]. Unfortunately, the underlying mechanism needs to be further explored to interpret this phenomenon.

### 4.4. Interaction Between Wood Metabolites and Mycobiota During the Initial Wood Decay

Our study revealed that ten metabolites were upregulated and sixteen metabolites were downregulated in the 120th-day groups compared to the first-day group (Figure 5a). For instance, lipids (Oleic acid) were upregulated in the 120th-day group. Instead, antibiotics (Chlortetracycline) were downregulated in the 120th-day group. To further investigate the decomposition mechanism of deadwood, altered metabolites were analyzed using the KEGG database for metabolic pathways. Phenylpropanoid biosynthesis was observed as the most significant pathway associated with the early stage of decomposition. Indeed, previous studies have shown that phenylpropanoid biosynthesis is an important metabolic pathway involved in the defense, growth, and development of pine trees [52]. Therefore, these selected metabolic pathways might be identified due to significant changes in their associated metabolites rather than being directly involved in the decomposition (Figure 5b). Meanwhile, it is challenging to distinguish host-derived metabolites from those produced by the microbial community in living trees [53,54]. However, the death of wood means the termination of trees’ physiological functions and life processes. Therefore, we speculate that the upregulated metabolites in the 120th-day group were largely influenced by microbial activities.

Previous studies have demonstrated that most of these downregulated metabolites are associated with the Tricarboxylic acid (TCA) cycle and its linked metabolic networks, including core TCA intermediates, TCA-related amino acids, and essential cofactors [55]. In contrast, the functional roles of the upregulated metabolites remain largely uncertain in deadwood. Our study indicated that the abundance of predominant fungi was strongly associated with up- or downregulated metabolites (Figure S4). For instance, as observed in correlation analysis, the abundance of *Agaricomycetes* and *Sordariomycetes*, dramatically increased from the first-day to 120th-day group, positively correlated with most upregulated metabolites but negatively correlated with all downregulated metabolites; meanwhile, the abundance of *Dothideomycetes* decreased from the first-day to 120th-day group, which is positively correlated with downregulated metabolites. These findings reveal close relationships between deadwood fungi and metabolites, suggesting that certain metabolites may serve as biochemical markers of fungal community dynamics.

## 5. Conclusions

Overall, this study has revealed the dynamics of microbiota in *Pinus massoniana* during initial wood decay, which may influence the subsequent colonization of the fungi. Our findings suggested that *Agaricomycetes* and *Sordariomycetes* rapidly established dominant status during the initial *Pinus massoniana* decay, and it was observed that most deadwood fungi might be directly recruited by the wood itself (living trees) rather than other sources during this period. The diversity of thick-barked deadwood fungi was significantly higher than in thin-barked deadwood. At the genus level, *Nectria*, *Cyberlindnera*, and *Kuraishia* were enriched in thick-barked *Pinus massoniana* deadwood, while these taxa might be related to consuming deadwood sugar. There were close relationships between dominate fungi and various metabolites, which might serve as biochemical markers of fungal communities’ dynamics. Monitoring pioneer microbial colonizers can facilitate predictions of wood decomposition trajectories, thereby providing a scientific basis for determining deadwood retention or removal cycles in forest management. In future studies, further relative contributions of various driving factors will be quantified to the overall understanding establishment process in deadwood fungal communities.

## Data Availability

The data that support the findings will be available in National Center for Biotechnology Information Sequence Read Archive under BioProject PRJNA1185929 following an embargo from the date of publication to allow for commercialization of research findings.

## Supplementary Materials

The following supporting information can be downloaded at: https://www.mdpi.com/article/10.3390/microorganisms13081743/s1, Table S1. Fungal differential ASVs between the thick and thin-barked groups in deadwood; Figure S1. Alpha diversity measures (mean Shannon diversity index and Simpson diversity index ± SE) of fungal communities in xylem; Figure S2. The data represent the relative abundance of top-15 xylem fungal community composition at the class level for a two decay time points, b two deadwood types, and c three sampling positions; Figure S3. The variable characteristics of xylem metabolites during the early stage of decomposition; Figure S4. Correlation heatmap.
