# Supplementary material for: Variation in Microbiota and Chemical Components Within Pinus massoniana During Initial Wood Decay
Source: Microorganisms. 2025 Jul 25;13(8):1743. doi: 10.3390/microorganisms13081743 (PMC12388206; doi:10.3390/microorganisms13081743)
Supplement: Supplementary file 1 [file microorganisms-13-01743-s001.zip › microorganisms-3723198-supplementary.pdf]

Table S1. Fungal differential ASVs between the thick and thin-barked groups in deadwood.

Thick vs. Thin	Level	Phylum	Class	Order	Family	Genus
ASV10	Enriched	*Ascomycota*	*Sordariomycetes*	*Hypocreales*	*Nectriaceae*	*Nectria*
ASV1411	Enriched	*Ascomycota*	*Sordariomycetes*	*Hypocreales*	*Nectriaceae*	*Nectria*
ASV26	Enriched	*Ascomycota*	*Saccharomycetes*	*Saccharomycetales*	*Phaffomycetaceae*	*Cyberlindnera*
ASV281	Enriched	*Ascomycota*	*Sordariomycetes*	*Hypocreales*	*Nectriaceae*	*Nectria*
ASV43	Enriched	*Ascomycota*	*Saccharomycetes*	*Saccharomycetales*	*Saccharomycetaceae*	*Kuraishia*
ASV44	Enriched	*Ascomycota*	*Sordariomycetes*	*Xylariales*	*Amphisphaeriaceae*	*Pestalotiopsis*
ASV476	Enriched	*Ascomycota*	*Sordariomycetes*	*Hypocreales*	*Nectriaceae*	*Nectria*
ASV5099	Enriched	*Ascomycota*	*Sordariomycetes*	*Hypocreales*	*Nectriaceae*	*Nectria*
ASV5913	Enriched	*Ascomycota*	*Sordariomycetes*	*Hypocreales*	*Nectriaceae*	*Nectria*
ASV5915	Enriched	*Ascomycota*	*Sordariomycetes*	*Hypocreales*	*Nectriaceae*	*Nectria*
ASV7890	Enriched	*Ascomycota*	*Sordariomycetes*	*Hypocreales*	*Nectriaceae*	*Nectria*
ASV8400	Enriched	*Ascomycota*	*Sordariomycetes*	*Hypocreales*	*Nectriaceae*	*Nectria*

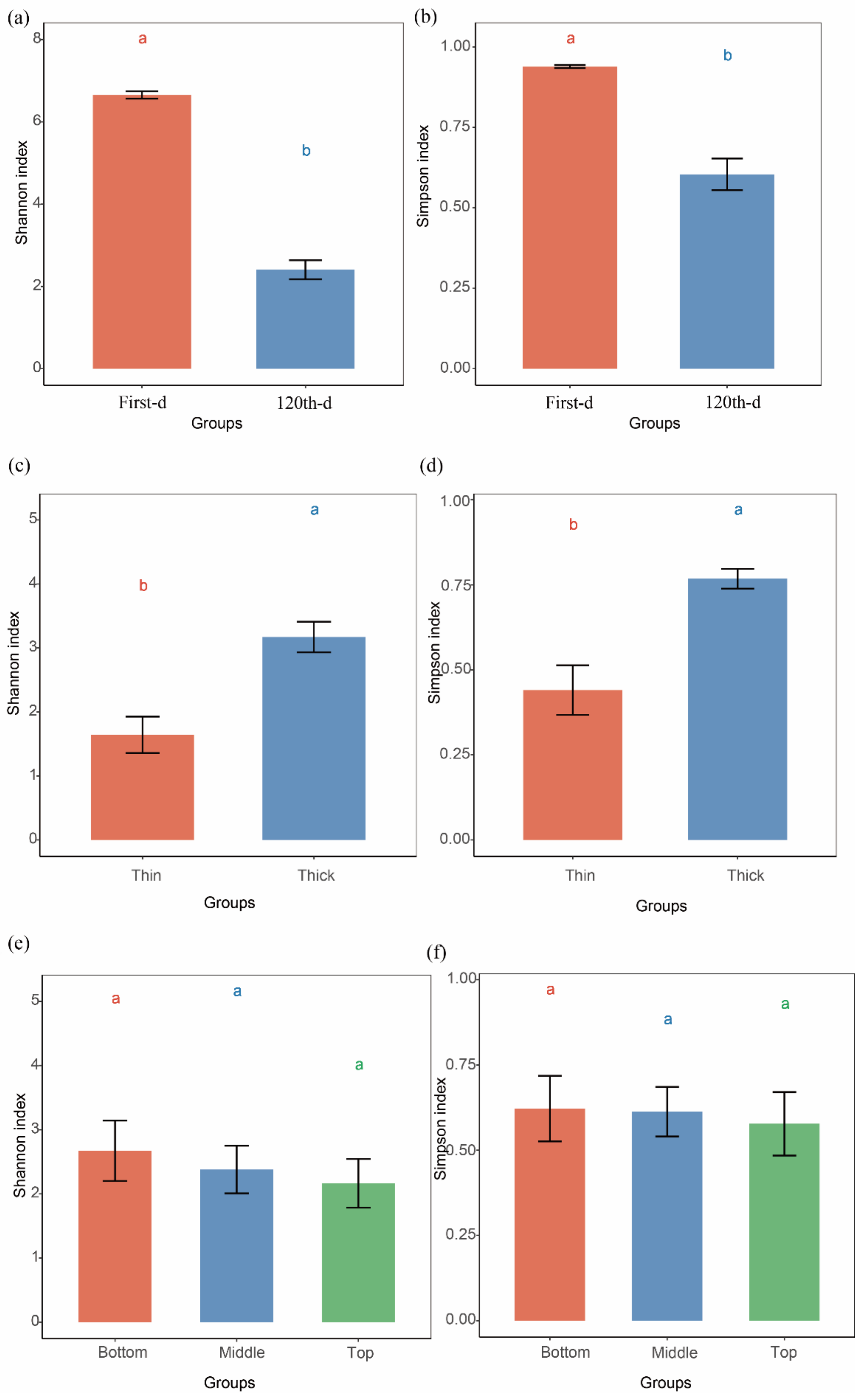

Figure S1. Alpha diversity measures (mean Shannon diversity index and Simpson diversity index ± SE) of fungal communities in xylem. Comparison of (**a**) Shannon diversity index, and (**b**) Simpson diversity index of xylem fungi between the two decay time points. Comparison of (**c**) Shannon diversity index, and (**d**) Simpson diversity index of xylem fungal community between the two deadwood types. Comparison of (**e**) Shannon diversity index, and (**f**) Simpson diversity index of xylem fungi among three sampling positions. Significant differences ($p < 0.05$) between conditions shown with letters at the top of the bar plot.

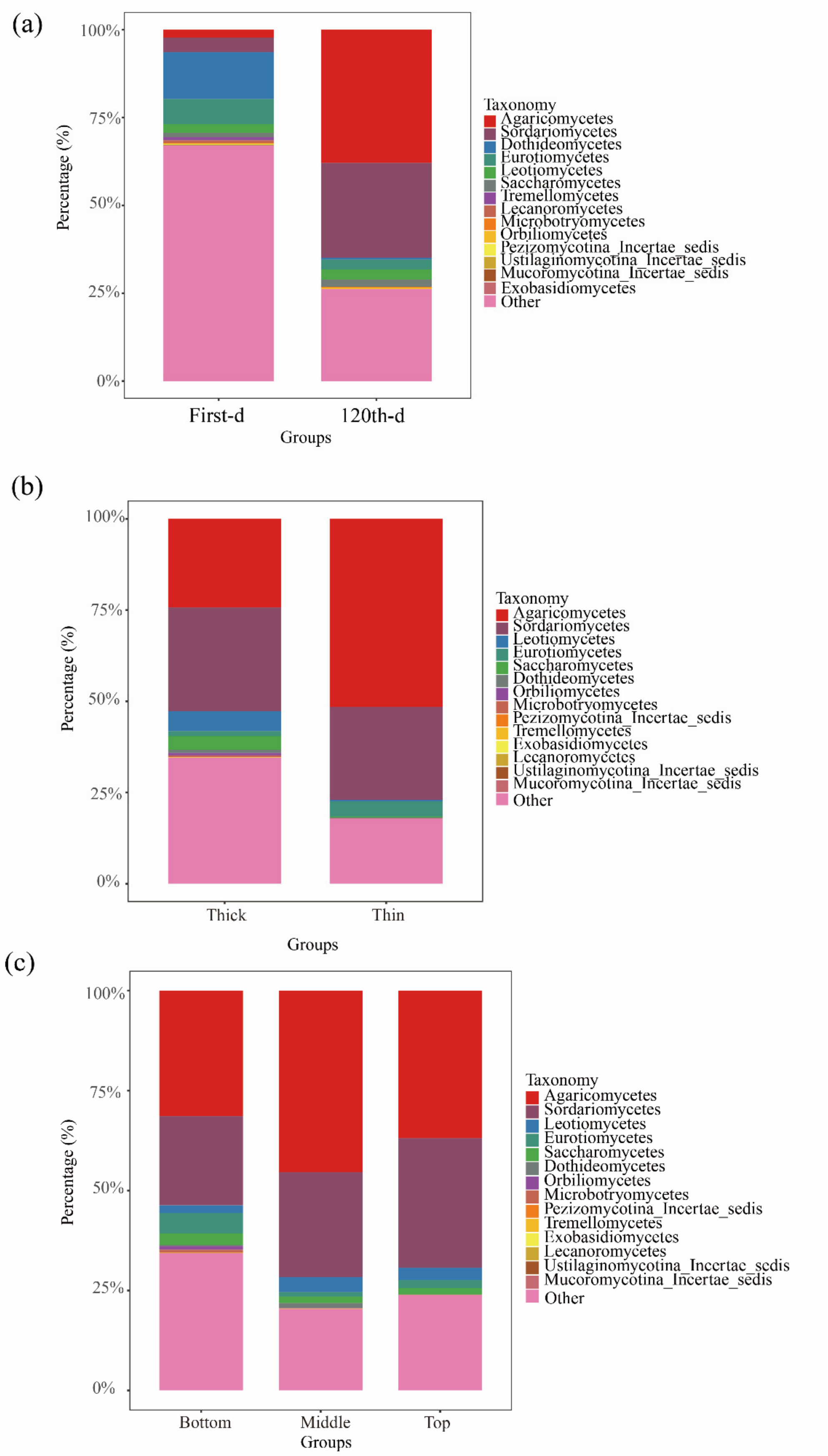

Figure S2. The data represent the relative abundance of top-15 xylem fungal community composition at the class level for (**a**) two decay time points, (**b**) two deadwood types, and (**c**) three sampling positions.

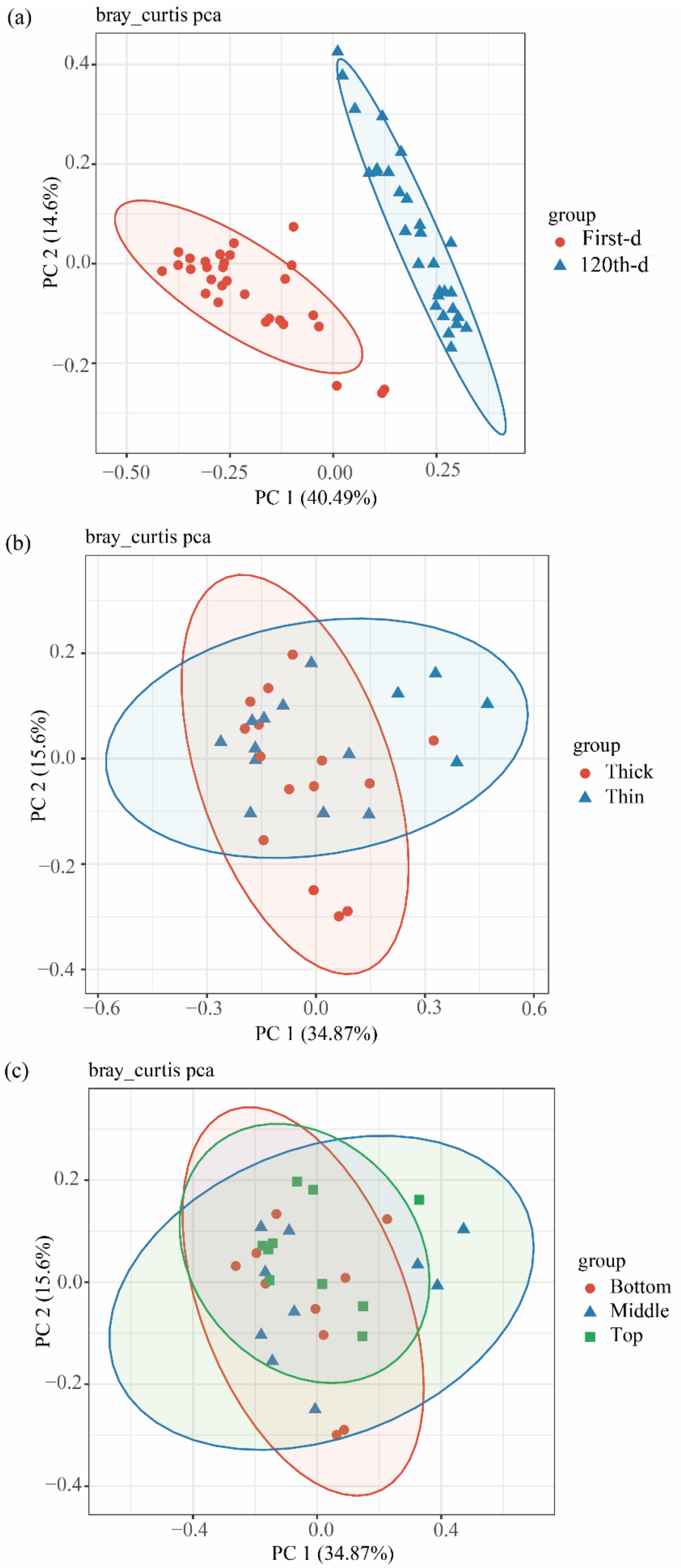

Figure S3. The variable characteristics of xylem metabolites during the early stage of decomposition. Principal component analysis (PCA) of the data of metabolites. Each point displays an individual sample. PC1 and PC2 represent the first and secondary principal components, respectively. The figures in brackets represent the explained variance ratio. (**a**) The groups of 1st-d and 120th-d are shown. (**b**) The groups of thick and thin on 120th-d are shown. (**c**) The groups of bottom, middle, and top are shown.

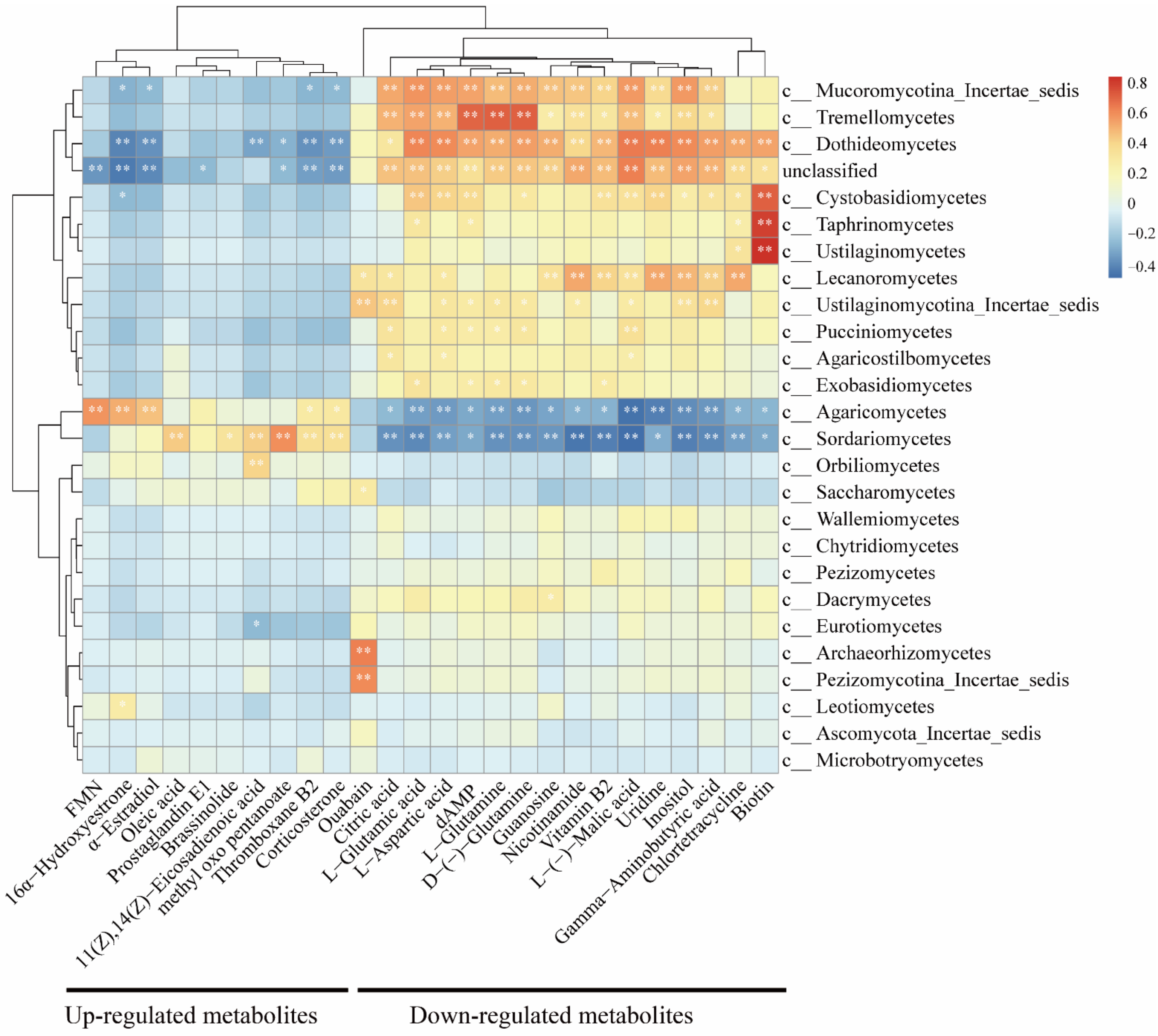

Figure S4. Correlation heatmap. Metabolites are presented on the x-axis, and microbes at the Class level are listed on the y-axis. The color bar shows the direction and magnitude of the correlation, with blue representing negative correlations and red showing positive correlations. Significance levels are denoted by asterisks: * $p < 0.05$; ** $p<0.01$.
